# Comprehensive analysis of cancer of unknown primary and recommendation of a histological and immunohistochemical diagnostic strategy from China

**DOI:** 10.1186/s12885-023-11563-1

**Published:** 2023-12-01

**Authors:** Min Ren, Xu Cai, Liqing Jia, Qianming Bai, Xiaoli Zhu, Xichun Hu, Qifeng Wang, Zhiguo Luo, Xiaoyan Zhou

**Affiliations:** 1 Department of Pathology, Fudan University Shanghai Cancer Center, 270 Dong’an Road, Shanghai, 200032 China; 2 Department of Oncology, Shanghai Medical College, Fudan University, Shanghai, 200032 China; 3 Institute of Pathology, Fudan University, Shanghai, 200032 China; 4 Department of Medical Oncology, Fudan University Shanghai Cancer Center, Shanghai, 200032 China

**Keywords:** Cancer of unknown primary, Clinicopathological feature, Immunohistochemical diagnosis, Molecular features, Survival

## Abstract

**Background:**

Previous studies on cancer of unknown primary (CUP) mainly focus on treatment and prognosis in western populations and lacked clinical evaluation of different IHC markers, so this study aimed to evaluate characteristics of CUP and recommend a diagnostic strategy from a single center in China.

**Methods and results:**

Data of 625 patients with CUP were retrospectively collected and reviewed. The patients ranged in age from 20 to 91 years, with a female-to-male ratio of 1.3:1. The predominant histological type was poor or undifferentiated adenocarcinomas (308; 49.3%). The results of Canhelp-Origin molecular testing for the identification of the tissue of origin in 262 of 369 patients (71.0%) were considered predictable (similarity score > 45), with the most common predicted primary tumor site being the breast (57, 21.8%). Unpredictable molecular results correlated with more aggressive clinical parameters and poor survival. Thee positivity rates of several targeted antibodies (GATA3, GCDFP15, TTF1, Napsin A, and PAX8), based on the clinically predicted site, were lower than those reported for the corresponding primary tumors. Nonetheless, TRPS1 and INSM1 were reliable markers of predicted breast carcinoma (75.0%) and neuroendocrine tumors (83.3%), respectively. P16 expression, as well as HPV and EBER testing contributed significantly to the diagnosis of squamous cell carcinomas. Survival analysis revealed that older ages (> 57), ≥ 3 metastatic sites, non-squamous cell carcinomas, bone/liver/lung metastases, unpredictable molecular results, and palliative treatment correlated with poor overall survival.

**Conclusions:**

We recommend a CUP diagnostic strategy involving the use of targeted antibody panels as per histological findings that is potentially applicable in clinical practice. The markers TRPS1, INSM1, and P16 expression, as well as HPV and EBER testing are particularly valuable in this aspect. Molecular testing is also predictive of survival rates.

## Introduction

Cancer of unknown primary (CUP), a biological enigma, is defined as histologically proven metastatic tumors without detectable primary tumors despite extensive and focused diagnostic investigations [1]. Several advancements in radiological, pathological, and molecular approaches have facilitated the identification of primary tumors in CUP patients, leading to a decrease in their proportion to 2–5% of all malignancies [2, 3]. However, the ambiguity surrounding the underlying etiology of this heterogeneous group, and the lack of effective therapies often results in poor prognosis, with a median overall survival (OS) of less than 12 months [2, 4].

Clinical intervention is theoretically determined by primary tumor type. Patients with CUP thus have few available therapeutic options and mostly receive empiric chemotherapy, including taxane- or platinum-based regimens with low response rates. Recently, several clinical trials have investigated the clinical value of molecular-guided site-specific treatment in CUP patients [5–7]. These studies have been based on the predominant hypothesis that the biology of CUP and response to treatment are similar to those of their predicted tissue of origin. Despite inconsistencies in published results, certain meta-analyses have demonstrated improved OS with site-specific treatment in comparison to that observed with empiric CUP treatment [8, 9]. Consequently, identification of the primary tumor in patients with CUP could guide treatment decisions, prolong survival time, and alleviate patient anxiety regarding extensive diagnostic procedures. Current clinical protocols for tracing the primary tumor in CUP, that involve physical examination, laboratory testing, radiological imaging, endoscopic examination, and histological as well as immunohistochemical (IHC) examinations, successfully identify it in only a small proportion of patients. The development of several molecular assays based on gene expression [10–12], DNA variations [13], DNA methylation [14, 15], and microRNA profiling [16] over the past few years have enabled the identification of the tissue of origin with outstanding accuracy that ranges from 60─90% [2], while these researches mainly based on primary or metastatic cases with known primary. Notably, the majority of such studies that primarily focus on treatment and prognosis have been conducted in western populations. Further, studies on histological and immunohistochemical diagnoses of patients with CUP in clinical practice is limited, especially in Chinese patients. In view of the differences in genetic alterations between different ethnicities, and the lack of clinical evaluation of different IHC markers in CUP, we aimed to comprehensively evaluate the clinical, pathological, immunohistochemical, molecular, therapeutic, and prognostic characteristics of patients with CUP, and further recommend a diagnostic strategy for CUP in clinical practice at a single center in China, in order to provide useful insights for the diagnosis and treatment of CUP patients.

## Materials and methods

### Patients

We retrospectively collected data from 625 CUP patients who were evaluated at the Fudan University Shanghai Cancer Center between May 2016 and March 2022. The study protocol was reviewed and approved by the Institutional Ethics Committee of Fudan University Shanghai Cancer Center. CUP patients were included based on the presence of histopathologically confirmed metastatic cancer without a detectable primary site despite extensive diagnostic investigations that included history, physical and laboratory assessments, pathology review, radiologic imaging and selective endoscopy [1]. The primary site was presumed on the basis of a comprehensive analysis of the above-mentioned investigations, and site-specific treatment was defined as the standard therapy for the alleged primary site. A majority of patients in the study were reviewed and analyzed by CUP multidisciplinary teams (MDTs) at Fudan University Shanghai Cancer Center.

### Immunohistochemical and molecular testing

All patients underwent IHC testing and the selection of different antibody panels including CK7, CK20, TTF1, PAX8, AE1/AE3, and P63, was made on the basis of tumor site, histology, and recommendation of the pathologist. Immunohistochemical studies were performed using an automated stainer (Benchmark XT; Ventana Medical Systems, Tucson, AZ, USA) as per the manufacturer’s instructions. Apart from IHC testing performed at initial diagnosis, immunohistochemical staining was carried out for TRPS1, INSM1, and P16 in 18 available predicted breast carcinomas, 4 neuroendocrine neoplasms, and 13 squamous cell carcinomas to explore their diagnostic value. The results of IHC testing were evaluated by an independent team of senior pathologists.

A total of 378 patients with complicated diagnoses, whose tumor tissues were available, underwent molecular testing. This was performed using the Gene Expression Test System for Human Tumor Origin Classification (Canhelp-Origin, approved by the National Medical Products Administration No.20,223,400,901, Canhelp Genomics Co., Ltd, Hangzhou, China). The testing strategy was designed to classify 21 common tumor types on the basis of gene expression profiles. Molecular testing and interpretation were performed as previously described [17, 18]. Briefly, total RNA was isolated from formalin-fixed paraffin-embedded (FFPE) tumor tissue samples using an FFPE Total RNA Isolation Kit (Canhelp Genomics, Hangzhou, China). Subsequent cDNA synthesis was performed using the High-Capacity cDNA Reverse Transcription Kit with RNase Inhibitor (Applied Biosystems, Foster City, CA, United States) as per the manufacturer’s instructions. Gene expression profiling was performed simultaneously in a 96-well plate using the Applied Biosystems 7500 Real-Time PCR System (Applied Biosystems). The classifier analyzed the gene expression patterns of 90 tumor-specific genes in each specimen, and generated similarity scores for each tumor type on the basis of the degree of similarity between the test specimen and the gene expression database. The similarity scores ranged from 0 (low similarity) to 100 (high similarity), and summed up to 100 across all 21 tumor types. The tumor type with the highest similarity score was presumed to be the primary tumor type that was predicted by Canhelp-Origin molecular testing.

Twenty-four patients underwent human papillomavirus (HPV) testing at the time of initial diagnosis. Further, six squamous cell carcinomas whose tumor tissues were available were evaluated for HPV status at the time of the present analysis for the identification of HPV-related cancers, including cervical or oropharyngeal cancers. Linear array HPV genotyping (Yaneng Bio, Shenzhen, China) was performed to detect 23 HPV types, including 17 high-risk HPV types (16, 18, 31, 33, 35, 39, 45, 51, 52, 53, 56, 58, 59, 66, 68, 73, and 82) and six low-risk HPV types (6, 11, 42, 43, 81, and 83). Additionally, in situ hybridization for EBV-encoded small RNA (EBER) was performed in 13 and 5 carcinomas with squamous cell differentiation at the time of initial diagnosis and present analysis, respectively, to identify Epstein-Barr virus (EBV)-related nasopharyngeal cancer.

### Statistical analysis and follow-up

Clinical and pathological characteristics including age, gender, number of metastatic sites (1, 2, ≥ 3), sites of metastases, histological diagnoses, immunohistochemistry, molecular testing, disease subgroups (favorable and unfavorable subgroups), and treatment (site-specific treatment, empiric chemotherapy, and palliative care) were summarized. Overall survival (OS) was measured from the date of diagnosis to either the date of death or last follow-up in surviving patients. The Kaplan-Meier method was used to determine survival curves, and differences between groups were tested using the log-rank test. Multivariate Cox proportional hazards models were used to identify factors predictive of survival, the results of which are expressed as hazard ratios (HR) and 95% confidence intervals (CI). Statistical analyses were performed using SPSS version 22.0 (SPPS, Inc.) and statistical significance was set at p < 0.05 (two-sided).

## Results

### Patient characteristics

The demographic details, clinicopathological features, and treatment data of the 625 patients with CUP included in this study are presented in Table 1. The patient ages ranged from 20 to 91 years (median, 57 years), and the female to male ratio was 1.3:1. A majority of patients presented with ≥ 3 metastatic sites (434, 69.4%), with the most common being the lymph node (424, 67.8%), followed by bone (159, 25.4%), lungs (132, 21.1%), and liver (98, 15.7%). The predominant histological types were poor or undifferentiated adenocarcinomas (49.3%), followed by well-to-moderately differentiated adenocarcinoma (20.5%), squamous cell carcinomas (19.5%), undifferentiated neoplasms (7.0%), neuroendocrine neoplasms (3.2%), and melanomas (0.4%). Endoscopy was performed in 443 (70.9%) patients. As per predefined criteria (2), 119 (19.0%) patients were categorized into the favorable subgroup and the remaining 506 (81.0%) were categorized into the unfavorable subgroup. In the context of treatment received, 275 patients were administered site-specific treatment, including chemotherapy and/or radiotherapy, 176 empiric chemotherapy, including taxane- or platinum-based regimens, 27 palliative care, and 147 did not receive any treatment.

**Table 1:** Clinicopathological features of 625 CUP patients

Characteristics	Patients N (%)	Characteristics	Patients N (%)
Age (years)		Immunohistochemistry	
Range (median)	20–91 (57)	CK7	448 (71.7)
< 40	53 (8.5)	CK20	357 (57.1)
40–50	93 (14.9)	TTF1	352 (56.3)
51–60	212 (33.9)	PAX8	329 (52.6)
> 60	267 (42.7)	CDX2	255 (40.8)
Gender		Disease subgroup	
Female	354 (56.6)	Favorable	119 (19.0)
Male	271 (43.4)	Unfavorable	506 (81.0)
Number of metastatic sites		Treatment	
1	118 (18.9)	Site-specific treatment	275 (44.0)
2	73 (11.7)	Empiric chemotherapy	176 (28.2)
≥ 3	434 (69.4)	Palliative care	37 (5.9)
Metastatic site		Unavailable	147 (23.5)
Lymph nodes	424 (67.8)	Results of Canhelp-Origin molecular testing	
Bone	159 (25.4)	Predictable	262 (41.9)
Lung	132 (21.1)	Unpredictable	107 (17.1)
Liver	98 (15.7)	Unable to analysis	9 (1.4)
Histology		Unavailable	247 (39.5)
Well-to-moderate adenocarcinoma	128 (20.5)	Predicted site of Canhelp-Origin molecular testing (N = 262)	
Poorly or undifferentiated adenocarcinoma	308 (49.3)	Breast	57 (21.8)
Squamous cell carcinoma	122 (19.5)	Gastroesophagus	33 (12.6)
Undifferentiated neoplasm	20 (3.2)	Lung	30 (11.5)
Neuroendocrine neoplasm	44 (7.0)	Cervix	26 (9.9)
Melanoma	3 (0.5)	Colorectum	21 (8.0)

### Immunohistochemical and molecular testing

IHC testing was performed on all patient samples. The most commonly used antibodies were CK7 and CK20, followed by TTF1, PAX8, and CDX2. While the IHC results provided diagnostic clues for the identification of primary tumor sites in 308 patients (49.3%), it was of no diagnostic value in 317 patients (50.7%). Further, 378 patients underwent the Canhelp-Origin molecular assay for identification of the primary tumor site, of which 369 patients received results that were eligible for analysis. The results of 262 patients were considered credible with a similarity score threshold of 45 or greater. The most commonly predicted primary tumor site was breast (57, 21.8%), followed by gastroesophagus (33, 12.6%), lung (30, 11.5%), cervix (26, 9.9%), colorectum (21, 8.0%), ovary (20, 7.6%), head and neck (19, 7.3%), urinary (14, 5.3%), neuroendocrine (12, 4.6%), liver/ cholangiocarcinoma (11, 4.2%), pancreas (10, 3.8%), kidney (4, 1.5%), mesothelioma (2, 0.8%), endometrium (2, 0.8%), and thyroid (1, 0.4%) (Table 1). A comparison of IHC and molecular testing in the 262 patients, revealed consistent results in 143, inconsistent results in 19, and results that could not be analyzed on account of IHC testing being of no diagnostic value in 100 patients. Statistical analyses revealed that unpredictable results (similarity score ≤ 45) in Canhelp-Origin molecular testing correlated better with the male gender, ≥ 3 metastatic sites, IHC testing of no diagnostic value, dedifferentiated histology, unfavorable disease subgroup, and poor survival, than that observed with predictable results (similarity score > 45) (Table 2).

**Table 2:** Comparison of clinicopathological features with predictable (similarity score > 45) and unpredictable (similarity score ≤ 45) Canhelp-Origin molecular testing results

Characteristics	Predictable molecular testing results (N = 262)	Unpredictable molecular testing results (N = 107)	p value
Age			
≤ 57	136	47	< 0.001
> 57	126	60	
Gender			
Female	145	41	< 0.001
Male	117	66	
IHC testing			
Diagnostic clues	149	46	< 0.001
No diagnostic value	113	61	
Histology			
Poorly differentiated carcinoma / Undifferentiated neoplasm	26	33	< 0.001
Adenocarcinoma / Squamous cell carcinoma / Other	236	74	
Disease subgroup			
Favorable	67	9	< 0.001
Unfavorable	195	98	

**Fig. 1:**
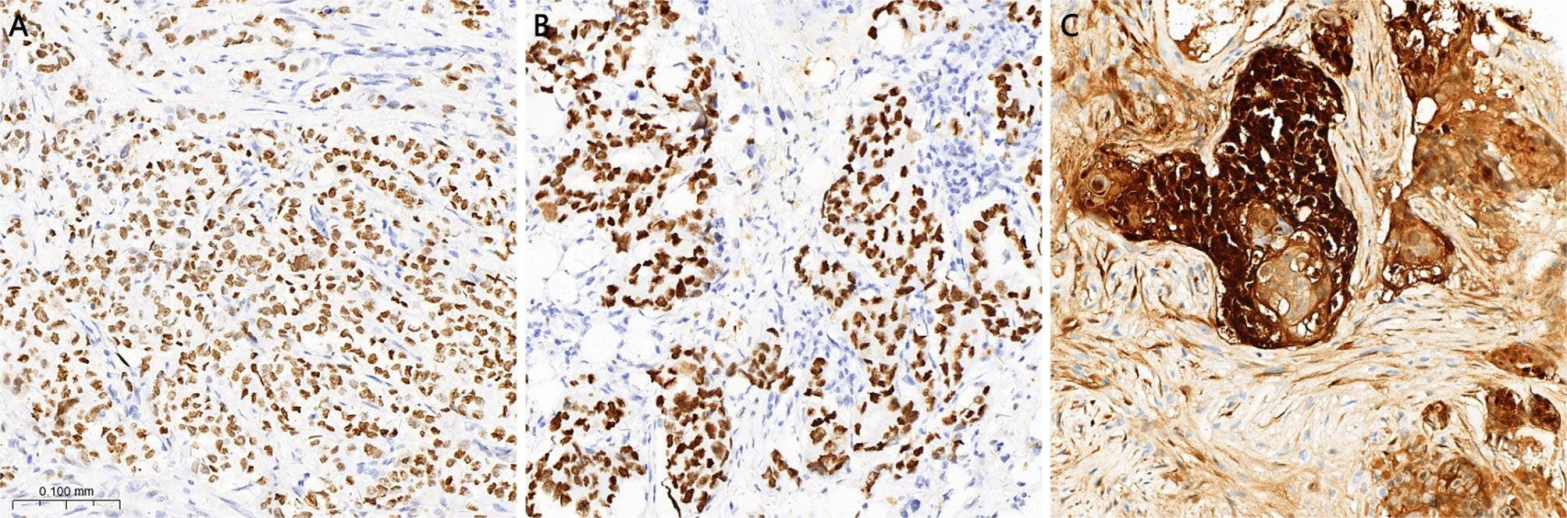
Representative immunohistochemical staining of TRPS1 **(A)**, INSM1 **(B)**, and P16 **(C)**

Based on the putative primary site predicted by the combination of clinical investigations, clinicopathological data and molecular testing, the sensitivity of representative antibodies in different CUP tumors was evaluated. As shown in Table 3, the sensitivity of most site-specific antibodies in the CUP tumors predicted to have a similar tissue origin than that for the corresponding primary tumors. These included GATA3, Mammaglobin and GCDFP15 in the breast; CDX2 and SATB2 in the colorectum; TTF1 and napsin A in the lung; PAX8, WT1, and ER in the ovary; and GATA3 in the urinary tract. The predicted primary sites in squamous cell carcinomas were mainly the head and neck, cervix, and gastroesophagus. Among these predicted head and neck (19), cervical (26), and gastroesophageal (7) squamous cell carcinomas, the rates of P16/HPV positivity were 40.0% (4/10), 92.9% (13/14), and 0% (0/6), respectively. Further, of eight head and neck, and ten cervical carcinomas tested, one head and neck carcinoma was EBER positive. Additionally, among the predicted neuroendocrine neoplasms, seven of 10 cases (70.0%) expressed the classical markers of neuroendocrine cell differentiation (Syn, CgA, and CD56), and four of five cases (80.0%) were INSM1 positive (Fig. 1).

**Table 3:** Positive rates of targeted diagnostic antibodies in different types of CUP predicted to have a similar tissue origin and corresponding primary tumor

Predicted primary tumor site^#^	IHC antibody	Positive rate in CUP tumor	Positive rate in primary tumor
Breast	GATA3	57.8% (26/45)	~ 90%^[32]^
	Mammaglobin	16.0% (4/25)	60-80%^[32]^
	GCDFP15	25.8 (8/31)	60%─80%^[32]^
	SOX10	69.2% (9/13)	~ 70%^[42]^
	TRPS1	75.0% (9/12)	~ 90%^[33]^
Colorectum	CK7	63.2% (12/19)	~ 40%^[43]^
	CK20	85.0% (17/20)	60─100%^[43]^
	CDX2	50.0% (6/12)	80─90%^[32]^
	SATB2	62.5% (5/8)	80─90%^[32]^
Gastroesophagus	CK7	90.9% (20/22)	40─70%^[43]^
	CK20	44.4% (8/19)	30─50%^[43]^
	CK19	88.9% (8/9)	~ 90%^[44]^
	CDX2	33.3% (5/15)	~ 30%^[32]^
Lung	CK7	88.0% (22/25)	> 90%^[43]^
	NapsinA	25.0% (3/12)	60–80%^[32]^
	TTF1	42.1% (8/19)	70─90%^[32]^
Pancreas	CK7	100.0% (10/10)	80─100%^[43]^
	CK19	100.0% (8/8)	~ 90%^[44]^
	SMAD4 loss	62.5% (5/8)	60%^[45]^
Ovary	PAX8	75.0% (12/16)	~ 90%^[32]^
	WT1	66.7% (8/12)	~ 90%^[32]^
	ER	23.1% (3/13)	~ 75%^[32]^
Urinary	GATA3	70.0% (7/10)	~ 80%^[32]^
	CK7	84.6% (11/13)	~ 80─90%^[43]^
Kidney	PAX8	100.0% (4/4)	> 80%^[32]^
	Vimentin	75.0% (3/4)	> 80%^[46]^

^#^Predicted primary tumor site was decided by the combination of clinical investigations, clinicopathological data and molecular testing

**Fig. 2:**
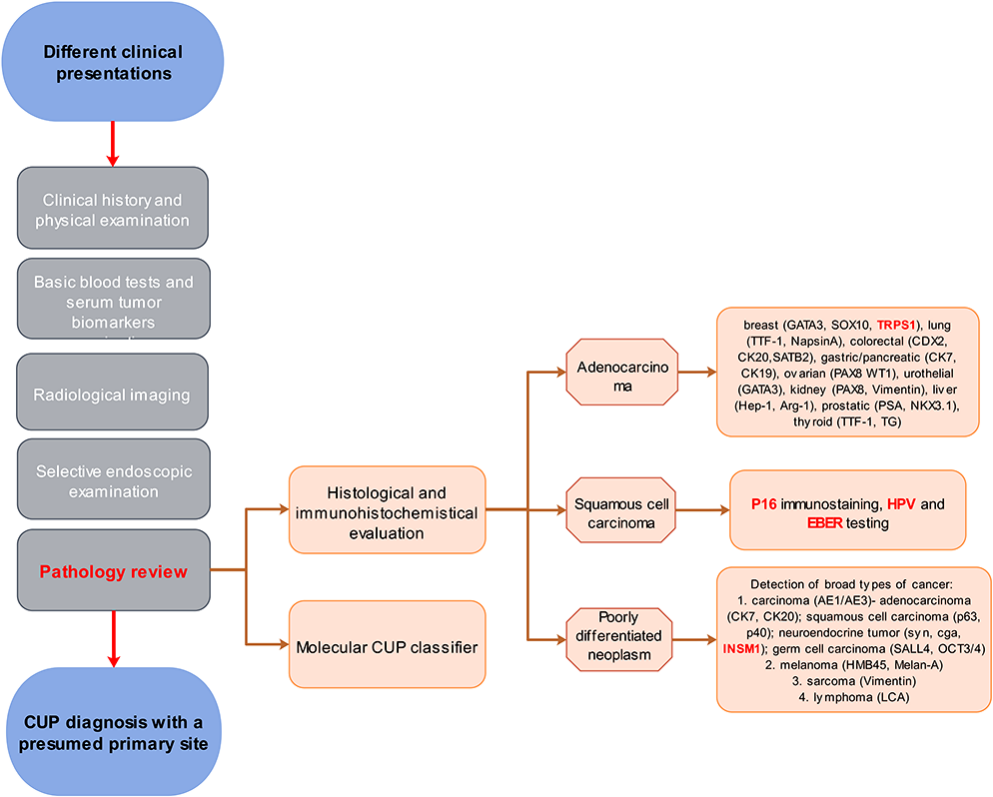
Recommended diagnostic strategy for CUP in clinical practice with emphasis on histological and immunohistochemical analysis

A considerable number of CUPs often lack the typical features characteristic of differentiation and the diagnostic performance of antibodies in our cohort and that reported in literature is unsatisfactory, leading to the failure of identification the culprit primary tumor by morphological and immunohistochemistry assessments. Therefore, molecular CUP classifiers are recommended to help provide clues about the primary tumor or predict the putative primary tumor type. Given the importance of conserving sufficient tissue for further molecular testing, we recommended a histological and immunohistochemical diagnostic strategy with superior sensitivity and performance for the identification of primary tumors in CUP. This involves the utilization of panels of targeted immunohistochemical antibodies, including TRPS1, INSM1, and P16 markers as well as HPV and EBER testing that is based on histological studies (Fig. 2). However, the IHC work-up should be individualized on the basis of the patients’ clinico-pathological features, including medical history, age, sex, site of metastasis, histological morphology, serum tumor markers, and radiology.

### Univariate and multivariate survival analyses

Follow-up data were available for a total of 498 patients. The follow-up duration ranged from 4 to 74 months (median, 29 months), and 292 (58.6%) patients had expired at the time of analysis. The median survival time was 14.8 months (range: 1–65 months), with a one and two-year survival rate of 53.3% and 37.3%, respectively.

**Fig. 3:**
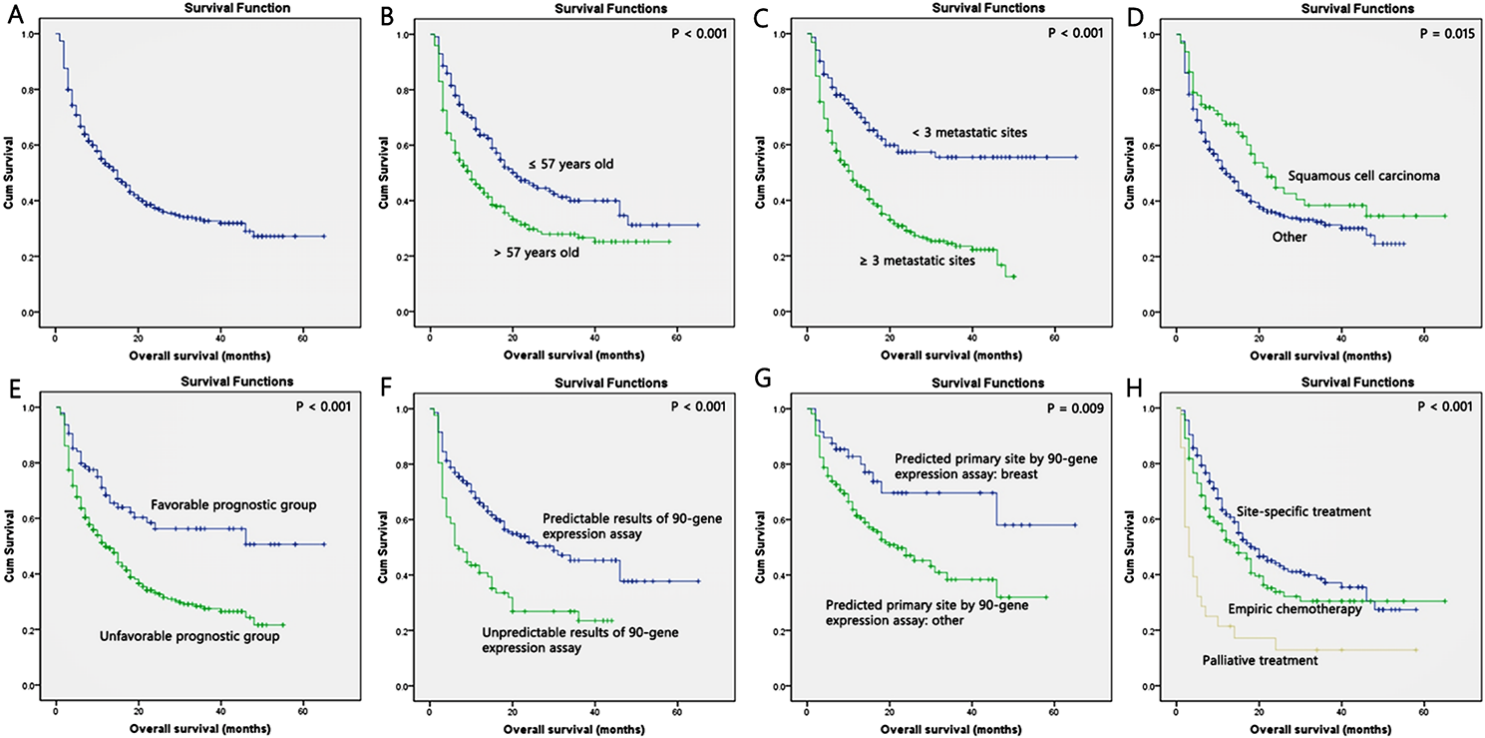
Kaplan-Meier overall survival curves for 498 CUP patients **(A)**. The overall survival curves of CUP patients by age **(B)**, number of metastatic sites **(C)**, histology **(D)**, disease subgroup **(E)**, Canhelp-Origin molecular testing results **(F)**, predicted site of Canhelp-Origin molecular testing **(G)**, and treatment **(H)**

Univariate analysis revealed a significant correlation between age (p < 0.001), number of metastatic sites (p < 0.001), histology (p = 0.015), disease subsets (p < 0.001), bone metastasis (p < 0.001), liver metastasis (p < 0.001), lung metastasis (p < 0.001), results of Canhelp-Origin molecular testing (p < 0.001), predicted primary site of Canhelp-Origin molecular testing (p = 0.009), and treatment (p < 0.001) with OS (Table 4; Fig. 3). Variables, including treatment and results of Canhelp-Origin molecular testing, that were incomplete for the cohort were excluded from further multivariate analyses. Multivariate analysis revealed better OS in patients aged ≤ 57 years (HR 0.646; 95%CI 0.509–0.819; p < 0.001) and with < 3 metastatic sites (HR 0.777; 95%CI 0.661–0.914; p = 0.002). In contrast, the presence of bone (HR 1.605; 95%CI 1.241–2.075; p < 0.001), liver (HR 1.456; 95%CI 1.082–1.960; p = 0.013), or lung metastasis (HR 1.323; 95%CI 1.018–1.720; p = 0.037) was found to be associated with poor OS (Table 4).

**Table 4:** Univariate and multivariate analyses of overall survival for 498 CUP patients

Characteristics	Patients N (%)	Univariate analysis		Multivariate analysis	
		Median Survival (Month)	p value	Hazard Ratio (95% CI)	p value
Age					
≤ 57	228	21	< 0.001	0.646 (0.509─0.819)	< 0.001
> 57	270	10		Reference	
Gender					
Female	222	18	0.061		
Male	276	12			
Number of metastatic sites					
< 3	151		< 0.001	0.777 (0.661─0.914)	0.002
≥ 3	347			Reference	
Histology					
Squamous cell carcinoma	96	22	0.015	1.017 (0.736─1.405)	0.918
Other	402	12		Reference	
Disease subgroup					
Favorable	95	/	< 0.001	0.744 (0.511─1.083)	0.122
Unfavorable	403	12		Reference	
Bone metastasis					
Present	132	6	< 0.001	1.605 (1.241─2.075)	< 0.001
Absent	366	20		Reference	
Liver metastasis					
Present	75	8	< 0.001	1.456 (1.082─1.960)	0.013
Absent	423	17		Reference	
Lung metastasis					
Present	109	11	< 0.001	1.323 (1.018─1.720)	0.037
Absent	389	16		Reference	
CK7 expression (N = 359)					
Positive	264	14	0.896		
Negative	95	11			
CK20 expression (N = 290)					
Positive	57	10	0.249		
Negative	233	12			
Result of Canhelp-Origin molecular testing (N = 300)					
Predictable	213	30	< 0.001		
Unpredictable	87	7			
Predicted site of Canhelp-Origin molecular testing (N = 213)					
Breast	48	/	0.009		
Other	165	22			
Treatment (N = 394)					
Site-specific treatment	229	19	< 0.001		
Empiric chemotherapy	137	15			
Palliative care	28	3			

## Discussion

Previous studies on CUP, a rare aggressive tumor have been primarily conducted in western populations and lack detailed evaluation of the clinical relevance of distinct IHC antibodies and molecular testing. The present study evaluated the clinical, pathological, immunohistochemical, molecular, and prognostic features of 625 CUP patients at a single center in China. Patients suspected of having CUP underwent comprehensive and standard diagnostic investigations, with more complicated cases being reviewed and discussed by the CUP MDT [19–21] which strengthened the credibility of our results. Consistent with previously reported results [21–23], the median age at diagnosis was approximately 60 years, with no significant differences attributable to patient gender. Histopathological analysis revealed that poorly or undifferentiated adenocarcinoma (49.3%) was the most common CUP subtype. Further, the proportion of squamous cell carcinoma (19.3%) while relatively higher in our cohort than that in Western populations (5–15%) [1, 22, 24, 25], was similar to that in other Asian populations [21, 26]. This is indicative of potential differences in CUP based on ethnicities. Lymph nodes, bone, liver, and lungs were found to be common metastatic sites, and most patients had multiple metastases at the time of diagnosis as has been previously reported [1, 27].

Identification of the primary tumor site is pivotal for the treatment and prognosis of patients with CUP. Pathological studies, the gold standard for tumor diagnosis, has limited performance in the identification of CUP primary sites [25, 28]. Various panels of IHC markers have been employed on the basis of morphological findings for the identification of the CUP primary sites, particularly in poorly differentiated or undifferentiated tumors. However, previous studies have revealed accurate identification by IHC analysis in only 50─65% of metastatic cancers [29, 30]. This suboptimal performance may be accounted for by the limited specificity and sensitivity of available markers [31]. Our results demonstrated no diagnostic value of IHC testing in the identification of primary tumors in approximately half of all patients. Further, the positivity rates of most site-specific antibodies in the CUP tumors predicted to have a similar tissue origin (for instance, GATA3 and GCDFP15 in breast, TTF1 and NapsinA in lung, and PAX8 and WT1 in ovary [32]) were lower than those reported for corresponding primary tumors. Apart from the traditionally employed IHC markers, a wide array of new IHC markers with improved performance for primary site prediction is now available. For instance, TRPS1, a highly sensitive and specific marker for breast carcinoma, especially for triple-negative breast carcinoma [33], was found to be a reliable marker for predicted breast carcinomas in our cohort (75.0%). Further, 70% of the predicted neuroendocrine neoplasms, a favorable CUP subgroup with specific treatments and good outcomes, were found to express the classical neuroendocrine markers, including Syn, CgA, and CD56. The sensitivity of INSM1 was found to be 80.0%, thus indicating its role as a valuable marker of neuroendocrine differentiation in both primary and metastatic neuroendocrine neoplasms [34, 35]. Common primary sites of squamous cell carcinomas of unknown primaries included the head and neck, lung, esophagus, and cervix. Previous studies have highlighted the importance of P16 expression, and HPV testing in the identification of oropharyngeal and cervical squamous cell carcinomas, and EBER testing in nasopharyngeal carcinomas [36–38]. We found a relatively high rate of P16/HPV positivity in predicted cervical carcinomas (92.9%), and head and neck carcinomas (40.0%). Based on our findings and available literature [32], we propose an effective diagnostic strategy for CUP, which involves the use of highly sensitive markers that target common primary tumor types, and preservation of sufficient tissue for further studies (Fig. 2). Because of relatively limited cases with available tissue sections during TRPS1, INSM1, P16/HPV testing, we especially investigated the diagnostic performance of these markers among recently suspected CUP cases. However, these patients lacked follow-up and treatment data and were therefore not included in the original analysis. Based on the primary site predicted by the combination of clinical investigations, clinicopathological data and molecular testing, TRPS1 expression was observed in 11 of 13 predicted breast carcinoma (84.6%), INSM1 expression was observed in all 3 predicted neuroendocrine neoplasms, and P16/HPV positivity was observed in all 4 predicted cervical carcinomas. Among 14 molecular-predicted head and neck squamous cell carcinomas, P16/HPV was positive in 4 cases who were further verified oropharyngeal squamous cell carcinomas. The above supplementary results partly verified the clinical value of TRPS1, INSM1, and P16 expression/HPV testing in the diagnosis of CUP, and we will accumulate more data to further explore their significance. However, in view of the still limited number of IHC markers and limited cases of some IHC markers in the present study, the clinical relevance of this approach needs further validation.

Due to the diagnostic dilemma of morphological and immunohistochemistry assessments in the identification of primary tumor, molecular CUP classifiers are recommended to help provide clues about the primary tumor or predict the putative primary tumor type. The use of advanced molecular assays has led to the identification of tissues of origin with approximately 80–90% accuracy in known tumors and prediction with 70% accuracy in CUP [4]. Canhelp-Origin molecular testing has been used to classify 21 tumor classes based on gene expression profiles and its clinical utility has been validated in a large cohort of cases comprising most of known primary tumors and a small portion of CUP [17, 18]. The predicted primary tumor sites from molecular and IHC testing in our cohort were highly concordant (88.3%, 143 out of 162). While 369 patients who underwent Canhelp-Origin molecular testing received reliable results, nearly 30% of CUPs had unpredictable results (similarity scores < 45), which is higher than that for non-CUP with a known primary [17]. These findings indicate the highly heterogeneous nature of genomic alterations that pose considerable challenges to the established and emerging molecular assays in clinical practice. In agreement with recent reports by Qi et al. [21] and Ye et al. [17], the common predicted primary sites in the Chinese population were the breast, gastroesophagus, and lungs. A further exploration of clinicopathological differences between patients with predictable and unpredictable molecular testing results revealed a correlation of the latter with more aggressive clinical parameters, including older age, dedifferentiated histology, unfavorable subgroups, and poor survival. Additionally, the survival of CUP patients with predicted breast cancer was significantly better than that of patients with other predicted tumor sites. This may be on account of most of such lesions presenting as isolated axillary nodal involvement, and their biology, treatment, and outcome being in accordance with stage II breast cancer [39]. The identification of primary sites and prediction of survival thus expands the potential clinical utility of molecular testing in patients with CUP.

The morbid prognosis of CUP is well documented in literature [40], with a median survival of less than one year, which is considerably worse than the results obtained in our cohort (median OS, 14.8 months). The relatively favorable outcome observed by us may be a consequence of the fact that the series was from a single cancer center with advanced medical expertise in China [27]. Moreover, the proportion of squamous cell carcinomas, typically associated with better OS [41], was higher than in our cohort. As previously reported, younger age, < 3 metastatic sites, squamous cell carcinoma, and favorable subgroups correlated with longer OS in our study [19, 23, 26, 41]. However, visceral (liver and lung) and bone metastases, as well as no oncological interventions were associated with shorter OS [21, 26]. The favorable prognosis of patients with site-specific treatment (median OS, 19 months) emphasizes the clinical significance of primary tumor identification in CUP. Thus, even though the prognosis of CUP remains dismal, early detection and site-specific or empirical treatment may potentially improve patient survival significantly.

Our study also has some limitations: For example, it is a retrospective and single-center study which may imply a potential selection bias. Moreover, only about 60% of CUP patients underwent Canhelp-Origin molecular testing in our cohort and nearly 30% of results were below the threshold, indicating that there is a need to further optimize the performance of the molecular testing. Finally, some limitations were present in the available tissue sections during TRPS1, INSM1, P16 staining and HPV testing, which could have decreased the statistical power of these results. And supplementary cases regarding these markers were not included in the overall analysis due to a lack of partial information.

In conclusion, the patients with CUP in our cohort were older, had multiple metastatic sites with common lymph node involvement, and predominantly poorly or undifferentiated adenocarcinoma. The proportion of squamous cell carcinoma among these patients was relatively higher than that reported in previous studies conducted in western populations. TRPS1 and INSM1 are sensitive markers for predicted breast and neuroendocrine neoplasms, respectively. P16 expression, HPV, and EBER testing have considerable diagnostic value in squamous cell carcinomas of unknown primaries. We propose a diagnostic strategy using highly sensitive markers including TRPS1, INSM1, and P16 expression, as well as HPV and EBER testing for identification of primary tumor sites in clinical practice. Canhelp-Origin molecular testing can identify the tissue of origin in most CUPs, and thus serve as a prognostic factor. The survival of patients with CUP remains poor in China, and further prospective studies are warranted to explore CUP biology and facilitate diagnosis and treatment.

## Data Availability

All data generated or analysed during this study are included in this published article.
